# Impacts of dietary supplementation of chitosan nanoparticles on growth, carcass traits nutrient digestibility, blood biochemistry, intestinal microbial load, and meat quality of broilers

**DOI:** 10.1093/tas/txae134

**Published:** 2024-09-14

**Authors:** Sheikh Adil, Ahmed K Aldhalmi, Manzoor A Wani, Irfan A Baba, I U Sheikh, Mohamed E Abd El-Hack, Nesreen Aljahdali, Najah M Albaqami, Dalia A Abuljadayel

**Affiliations:** Division of Livestock Production and Management, Faculty of Veterinary Sciences & Animal Husbandry, SKUAST-K, Srinagar, India; College of Pharmacy, Al-Mustaqbal University, Babylon, Iraq; Division of Livestock Production and Management, Faculty of Veterinary Sciences & Animal Husbandry, SKUAST-K, Srinagar, India; Division of Livestock Production and Management, Faculty of Veterinary Sciences & Animal Husbandry, SKUAST-K, Srinagar, India; Division of Livestock Production and Management, Faculty of Veterinary Sciences & Animal Husbandry, SKUAST-K, Srinagar, India; Poultry Department, Faculty of Agriculture, Zagazig University, Zagazig, Egypt; Department of Biological Science, College of Science, King Abdulaziz University, Jeddah, Saudi Arabia; Department of Biological Sciences, Faculty of Sciences, King Abdulaziz University, Jeddah, Saudi Arabia; Department of Biological Sciences, Faculty of Sciences, King Abdulaziz University, Jeddah, Saudi Arabia

**Keywords:** broilers, chitosan nanoparticles, performance, blood health, meat quality

## Abstract

This study explores the impact of chitosan nanoparticles (**CNP**) on the performance, nutrient digestibility, blood biochemical, immunity, microbial load, carcass traits, and meat attributes of broilers. A total of 200 7-d-old Cobb chicks were distributed to 4 groups, each replicated 5 times, with 10 birds in each replicate. The experimental diets were as follows: First group was fed a basal diet only (control); 2nd, 3rd, and 4th groups received a basal diet supplemented with 0.2, 0.3, and 0.4 g CNP/kg of feed, respectively. Results showed that the body weight (**BW**) and body weight gain significantly improved (*P* < 0.05) in the birds belonging to the 0.4 CNP group compared to the other groups. The best feed efficiency (feed conversion ratio [**FCR**]) was found in the group supplemented with a 0.4-g CNP/kg diet. The digestibility coefficients for dry matter and crude protein were significantly higher, and ether extract was significantly lower in the 0.4 g CNP/kg group than in other groups (*P* < 0.05). Broiler birds of the 0.4 CNP group had significantly (*P* < 0.05) reduced serum cholesterol, AST, and ALT levels. The humoral immunity (increased serum IgG and IgM levels) tended to improve in birds fed 0.3 and 0.4 g CNP/kg of feed. Compared to the control, total bacterial load and coliform count decreased significantly (*P* < 0.05) by supplementing 0.4 g CNP in the diet. The dressing weight, breast weight, and abdominal fat % were altered in birds receiving dietary 0.4 g CNP/kg. The treatment with CNP at 0.4 g/kg feed enhanced the broiler meat quality by increasing the values for water holding capacity, ABTS [2, 2ʹ-azinobis (3-ethylbenzothiazoline-6-sulfonic acid)], DPPH (2,2-diphenyl-1-picrylhydrazyl) while reducing the thiobarbituric acid reactive substances (TBARS) value. Based on the results above, it could be concluded that CNP supplementation at 0.4 g/kg is recommended as a beneficial feed additive for broiler chickens.

## Teaser

This work examined how adding chitosan nanoparticles (**CNP** ) to the diet affected performance, nutrient digestibility, blood biochemical, immunity, microbial load, carcass traits and meat attributes of broilers. Findings suggested that CNP enhances growth, strengthens the immune system, improves meat quality, and contributes to the overall health of the hens.

## Introduction

Feed supplements are commonly used to boost poultry’s health and production efficiency, producing high-quality meat and eggs (Gerber et al., 2007; Mahasneh et al., 2024). Conventionally, antibiotic growth promoters (**AGPs**) have been employed in poultry diets for decades to enhance production and control pathogenic microbes (Shao et al., 2021; Swaggerty et al., 2022). However, microbial pathogens have developed resistant strains due to the excessive and unselective utilization of AGPs (Anusuya et al., 2012; Farooq et al., 2013), leading to residues in animal products and probable transmission of resistant strains to humans through the food chain (Zhang et al., 2022). Due to these public health concerns and consumer pressure, several antibiotic substitutes that can stimulate growth and disease resistance and sustain animal health have been utilized. These include prebiotics (Yaqoob et al., 2021), organic acids (Adil et al., 2010), prebiotics (Anadon et al., 2019; Abd El-Hack et al., 2020, 2021), essential oils and plant extracts (Adil et al., 2020), enzymes (Yousuf et al., 2012), aquatic plants (Ara et al., 2018; Zaffer et al., 2021), etc. Among natural products, chitosan is one of the biopolymers less commonly sourced as a feed supplement (Amirthalingam et al., 2021). It is the second most plentifully available biodegradable material after cellulose, derived from chitin by deacetylation (Amirthalingam et al., 2021).

Chitosan is a safe and eco-friendly material with promising benefits when incorporated as a feed additive in animal feeding and its major sources are crustacean shells, the cell wall of fungi, and the exoskeleton of insects (Adewuyi et al., 2019; Jacaúna et al., 2021; Algothmi et al., 2024). Chitosan has been found to have antimicrobial, antioxidant, immunomodulatory, antitumor, anticoagulant, and anticholesterolemic properties (Zou et al., 2019; Azeem et al., 2022; Maleki et al., 2022).

Dietary supplementation of chitosan improves animal growth indices (Xu et al., 2014), immunity and suppresses intestinal pathogenic microbes in fish (Abdel-ghany and Salem, 2020). As a feed additive in the broiler diet, chitosan tends to decline the colonization of foodborne pathogens like *Salmonella typhimurium* (Kong et al., 2010; Menconi et al., 2014). It also has potent immunostimulatory activity by enhancing the serum amounts of immunoglobulins like IgG, IgM, and IgA (Li et al., 2016), increasing the total leucocyte count (Nuengjamnong and Angkanaporn, 2017) and beginning the release of chemokines, cytokines, etc. (Fong and Hoeman, 2018). The hypocholesterolemic effects of chitosan may be due to its adverse effects on circulating adipocytokines, preventing lipid accumulation in muscle (Xu et al., 2014). Several nanoform particles have been previously used to support poultry birds’ performance and production parameters. Chitosan nanoparticles (**CNP**), because of their nano size (<100 nm), can depict potent antimicrobial, antioxidant, and immunomodulatory activities in addition to enhancing growth performance and microbial balance in the gut (Divya and Jisha, 2018; Xu et al., 2018). The present study hypothesized that CNP may positively affect broilers’ growth criteria and health status. Therefore, the current investigation was intended to estimate the efficacy of CNP as feed additives in broiler chicken on performance, nutrient digestibility, serum biochemistry, immune response, microbial load, carcass traits, and meat quality.

## Materials and Methods

### Chitosan, Nanoparticles, and Morphology

**Figure 1.**
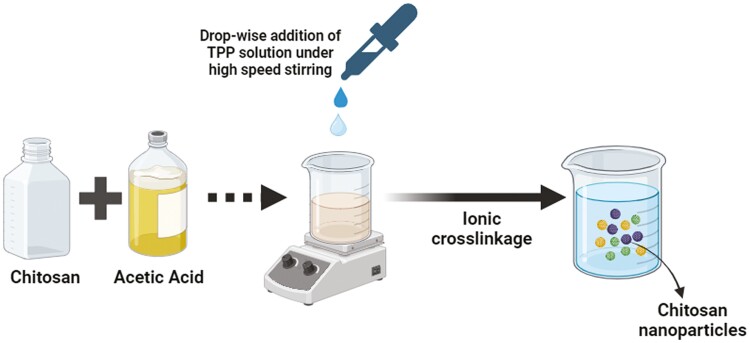
Schematic diagram illustrating the formation of chitosan nanoparticles.

The chitosan (derived from crab shell) was purchased from HiMedia Laboratories Pvt. Ltd, India, and sodium triphosphate pentabasic (**TPP**) from Sigma-Aldrich. The chitosan nanoparticles were prepared using the ionic gelation method (Fig. 1) described by Iswanti et al. (2019). This method is established on electrostatic interfaces between the positively charged chitosan biopolymer and negatively charged TPP. The morphology of chitosan nanoparticles was estimated by Scanning Electron Microscopy (**SEM**; Hitachi 3600 N model) having a 5-axis motorized phase connected with an ultra-dry compact EDS detector (Thermo Scientific).

### Animals, Design, and Diets

The study used 200 unsexed broiler chicks at 7 d of age with an average body weight (**BW**) of 152 ± 2.46 g. Chicks were randomly distributed on floor pens among groups containing 40 birds (5 replicates of 10 birds). The dietary treatments were as follows: the first control group was fed a basal diet only, whereas the 2nd, 3rd, and 4th groups received the basal diet supplemented with 0.2, 0.3, and 0.4 g of CNP/kg diet. The basal diets (Table 1) based on corn–soybean were formulated as per the requirements of BIS (2007). Chickens had free access to feed and water throughout the trial period. The light was supplied 24 h for the first 3 d, followed by a decrease of 1 h each day till it reached 18 h. To provide thermo-comfort to the birds, the house temperature was set at 95 °F and reduced by 5 °F each week. The study was conducted at the experimental farm of the Division of LPM, SKUAST-Kashmir for 42 d. It was approved by the Institutional Ethics Committee of FVSc & AH, SKUAST-K.

**Table 1.** Composition and chemical analysis of the basal diet

Ingredients	Starter (1 to 3 wk)	Finisher (4 to 5 wk)
Maize	56.45	59.71
Soybean meal	32.93	31.27
Fish meal	4.19	1.94
Soybean oil	3.38	3.87
Limestone	0.80	0.81
Di calcium phosphate	1.40	1.63
Salt	0.30	0.30
DL-Meth	0.13	0.11
Lysine	0.06	0.00
Trace mineral premix^1^	0.10	0.10
Vit Premix^2^	0.15	0.15
B comp	0.02	0.015
Ch. Chloride	0.05	0.05
Toxin binder (aluminosilicate)	0.05	0.05
Chemical analysis^*^		
Crude protein	21.60	20.01
Metabolizable energy, kcal/kg	3,079.76	3,134.73
Calcium %	1.01	0.99
Available P %	0.46	0.45
Methionine + cystine	0.985	0.896
Lysine %	1.20	1.05
Methionine %	0.51	0.45

^1^Trace mineral premix (mg/kg diet): Mg 300, Mn 55, I 0.4, Fe 56, Zn 30, and Cu 4.

^2^Vitamin premix (per kilogram diet): vitamin A 8,250 IU, vitamin D3 1,200 ICU, vitamin K 1 mg, vitamin E 40 IU, vitamin B1 2 mg, vitamin B2 4 mg, vitamin B12 10 µg, niacin 60 mg, pantothenic acid 10 mg, choline 500 mg.

^*^Calculated according to BIS (2007).

### Growth Performance and Carcass Traits

Feed intake (**FI**) and BW were documented at the 1st, 3^rd^, and 6th weeks of age. Body weight gain (**BWG**) and feed conversion ratio (**FCR**) were calculated for 1 to 3, 4 to 6, and 1 to 6 wk. At 6 wk of age, 10 birds per treatment were randomly chosen, weighed, and slaughtered ethically for carcass evaluation study. Dressed percentage, breast, thigh, heart, liver, gizzard, and abdominal fat percentage were recorded. The breast samples were taken from birds for meat quality analysis.

### Digestibility Trial

The digestibility trial was performed at 35 d of age and lasted for 4 d (35th to 38th day). Metabolic cages and 2 chickens per replicate were chosen randomly. The digestibility of ether extract (**EE**), dry matter (**DM**), crude protein (**CP**), crude fiber (**CF**), and phosphorous (**P**) was estimated by AOAC (2005) and calcium (**Ca**) by Talpatra et al. (1940). The total feed consumed by each group of birds in the separate dietary treatments was documented. The droppings emptied over the same time were collected quantitatively dried for 4 to 5 days in an oven at 60 ± 5 °C until a regular weight was reached. The representative excreta samples and test diets were ground and stored in airtight containers for further assessment.

The consumption excreted and maintained amounts of DM, nitrogen, Ca and P were estimated in grams per bird per day basis, and percent (%) retention was considered based on total intake by the following formula:


$$\begin{array}{lll} Nutrient & \;retention\;\%\; & \;=\frac{\left(Nutrient\;intake-Nutrient\;in\;faeces\right)}{Nutrient\;intake}\;\times \;100 \end{array}$$

### Blood Biochemical

Blood samples were taken from the slaughtered birds in vacutainers. Following coagulation, the centrifugation was done at 3,000 rpm for 30 min, and the serum was harvested and stored at −20 °C until analysis. Various biochemical parameters were estimated like total protein (**TP**), cholesterol (**Chol**), triglycerides (**TG**), albumin (**Alb**), globulin (**Glob**), liver function enzymes such as aspartate aminotransferase (**AST**), and alanine aminotransferase (**ALT**), with the help of auto-analyser (Csense 200^CD^ Medsource Ozone Biomedicals Pvt. Ltd) utilizing respective biochemical kits from the same manufacturer.

### Immune Response

The serum IgG and IgM immunoglobulins were assessed using Elisa Kits (Shanghai Coon Koon Biotech, China). Regarding anti-SRBC titer, 2 birds per replicate at 35 d of age were injected with 0.1% of sheep red blood cells (**SRBC**) @ 0.1 mL/kg BW into the wing veins. The injected birds were distinguished from other birds by markings. At 42 d of age, blood samples were taken from the injected birds, and serum was harvested and stored until analysis was performed. An anti-SRBC antibody titer was recorded using a microhaemagglutination assay utilizing 96-well microplates, as explained by Akhlaghi et al. (2013).

### Microbial Load

From the slaughter birds, caecal samples were collected, diluted, and homogenized in 90 mL sterilized peptone water, and serial 10-fold dilutions were made. Total bacterial count (**TBC**), *Lactobacilli* and *Coliform* counts were done using Plate count, de Man Rogasa and Sharpe, and McConkey media, respectively, as per the procedure described by Speck (1984) and results were expressed as log_10_ CFU/g.

### Meat Quality

Physical traits like pH, water holding capacity (**WHC**) and drip loss (**DL**) were calculated. At 24-h postslaughter, the pH values of the breast meat were measured as per Gökalp et al. (2001), utilizing a pH meter (TANCO India). The WHC of meat samples was assessed by mixing 10 g minced meat samples in 15 mL of 0.6 M NaCl for 2 min followed by refrigerated (4 °C) holding for 15 min. The sample was then shuddered and centrifuged at 5,000 rpm for 15 min and the supernatant fluid was transferred and determined (Brondum et al., 2000). The frozen meat samples’ initial weight (W1) was measured and recorded to determine the DL. The samples were placed in labeled polyethylene bags and suspended at 4 °C for 24 h. Afterward, the meat samples were weighed again, and the final weight (W2) was documented. The calculation of DL was performed using the equation provided below:


$$\begin{array}{l} Driploss\;\left(\;\%\;\right)=\frac{(W1-W2)}{W1}\;\times \;100 \end{array}$$

The color coordinates [redness (*a*), lightness (*L*), and yellowness (*b*)] of the cross-sectional areas of breast samples were measured directly on the surface of the muscle using a colorimeter (YS3060, China).

Lipid oxidation was assessed using a Thiobarbituric Acid Reactive Substance (TBARS) assay. The approach of Witte et al. (1970) was used to estimate the TBARS value with minor changes. For 2 min, 10 g of material was triturated in a 2 M orthophosphoric acid solution with 25 mL of pre-cooled 20% trichloroacetic acid (**TCA**). The substances were quantitatively moved into a beaker after rinsing with 25 mL of cooled distilled water. After adequate mixing, the contents were filtered via ash-free filter paper (Whatman filter paper No. 1 provided by GE Healthcare U.K.). In test tubes, 3 mL of TCA extract (filtrate) was combined with 3 mL of TBA (thiobarbituric acid) reagent (0.005 M) and left in the dark for 16 h. A 3 mL of 10% TCA and 3 mL of 0.005 M TBA reagent were combined to make a blank sample. The UV-VIS spectrophotometer was applied, and the absorbance (O.D.) was determined at a fixed wavelength of 532 nm (HITACHI, UV-Spectrophotometer U-1800). By multiplying the O.D. value with the *k* factor 5.2, the TBARS value was measured as milligram malondialdehyde per kilogram of sample.

The antioxidant capacity of breast meat samples was evaluated by ABTS [2, 2-azinobis (3-ethylbenzothiazoline- 6-sulfonic acid)] and DPPH (2, 2-diphenyl-1-picrylhydrazyl) assay. A 5 g meat sample was triturated in 20 mL ethanol for 2 min, filtered through Whatman filter paper no. 42. For ABTS assay, to 1 mL filtrate, 2 mL ABTS working solution (7 mM) was added and absorbency was measured using a spectrophotometer (Hitachi, U-1800) at a wavelength of 734 nm after 20 min (Shirwaikar et al., 2006). For DPPH assay, 1 mL filtrate, 1 mL 0.1 M Tris-HCL buffer (pH 7.4), and 1 mL DPPH reagent (250 µM) were added. The absorbency was measured immediately (At_0_) and after 20 min (At_20_) by spectrophotometer (Hitachi, U-1800) at a fixed wavelength of 517 nm (Saleh et al., 2018).

Calculations were done as follows:


$$\begin{array}{l} ABTS\;activity\left(\;\%\;inhibition\right)\;=\;\left[\frac{\left(0.7\;\text{-}\;At_{20}\right)}{0.7}\right]\times 100 \end{array}$$

$$\begin{array}{l} DPPH\;activity\left(\;\%\;inhibition\right)=100-\left(\frac{At_{20}}{At_{0}}\right)\times 100 \end{array}$$

### Statistical Analysis

Statistical software, SPSS, version 20.0, was used to analyze data through one-way ANOVA. The differences in the means were considered significant at *P* < 0.05 and Duncan’s multiple range test was used to test the significance between means. The model that was employed was


$$\begin{array}{l} Y_{ij}\;=\mu +\;T_{i}\;+\;e_{ij} \end{array}$$

where *Y*_*ij*_ is an observation treatment, μ is an overall mean, *T*_*i*_ is the *i*-treatment effect, and *e*_*ij*_ is the random error.

## Results

### Scanning Electron Microscopy

**Figure 2.**
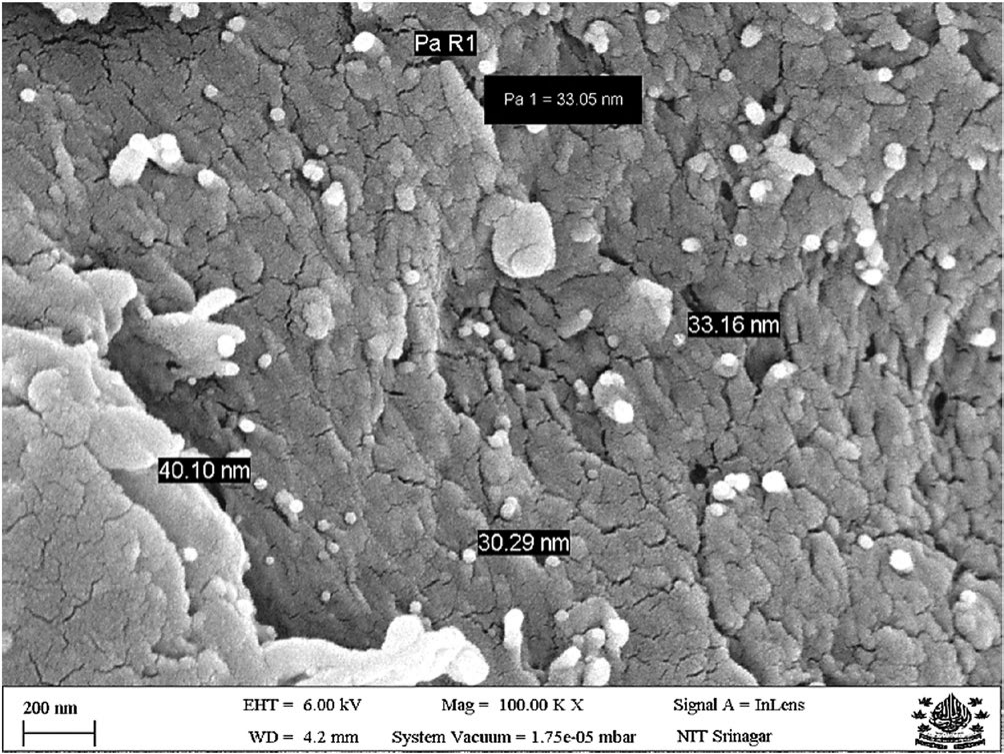
Scanning electron microscopy of chitosan nanoparticles.

The morphology of chitosan nanoparticles, as depicted by scanning electron microscopy, is shown in Figure 2. The results revealed that the chitosan nanoparticles had rough surfaces, were almost spherical, and accumulated in some places. Further, most of the particles are smaller than 100 nm.

### Growth Performance

The influence of CNP supplements on the growth performance of birds is given in Tables 2 and 3. The supplementation of CNP in the diet significantly (*P* < 0.05) affected broiler growth performance in improved BW and BWG. Better performance was observed in the birds provided with 0.4 g of CNP/kg in feed. FI was not altered (*P* > 0.05) by any level of CNP supplementation compared to the control. Broiler birds fed 0.4 g of CNP/kg supplemented diet showed better FCR than other groups and control.

**Table 2.** Effects of dietary CNP supplementation on BW and BWG of broilers

Attributes	CNP (g/kg diet)^*^				SEM	*P*-value
	0	0.2	0.3	0.4		
Body weight (g)						
1 wk	154.93	155.11	153.69	155.28	0.97	0.947
3 wk	683.02^b^	687.34^b^	692.53^ab^	703.88^a^	2.89	0.048
6 wk	1,966.00^b^	1,978.54^b^	1,986.85^b^	2,019.82^a^	5.49	<0.001
Body weight gain (g)						
1 to 3 wk	528.09^b^	532.23^ab^	538.83^ab^	549.20^a^	3.22	0.037
4 to 6 wk	1,283.19^b^	1,297.74^ab^	1,311.69^ab^	1,322.87^a^	5.69	0.048
1 to 6 wk	1,811.27^b^	1,829.98^ab^	1,850.52^ab^	1,872.07^a^	6.58	0.001

BW: body weight; BWG: body weight gain; CNP: chitosan nanoparticles.

^*^The average of 5 pens of 10 birds per pen.

The means in the same column bearing different superscripts are significantly different at *P* < 0.05.

**Table 3.** Effects of dietary CNP supplementation on FI and FCR of broilers

Attributes	CNP (g/kg diet)^*^				SEM	*P*-value
	0	0.2	0.3	0.4		
Feed intake (g)						
1 to 3 wk	636.69	635.86	640.60	659.60	4.18	0.144
4 to 6 wk	2,525.51	2,534.34	2,541.80	2,533.56	7.92	0.926
1 to 6 wk	3,162.20	3,170.20	3,182.40	3,193.16	7.62	0.528
Feed conversion efficiency						
1 to 3 wk	1.21	1.20	1.19	1.20	0.003	0.187
4 to 6 wk	1.97^b^	1.95^b^	1.94^ab^	1.92^a^	0.010	<0.001
1 to 6 wk	1.74^b^	1.73^b^	1.72^ab^	1.71^a^	0.007	<0.001

CNP: chitosan nanoparticles; FI: feed intake; FCR: feed conversion ratio.

^*^The average of 5 pens of 8 birds per pen (6 wk; day 42).

The means in the same column bearing different superscripts are significantly different at *P* < 0.05.

### Digestibility Trial

Table 4 shows the nutrient digestibility results for broilers in response to supplementation with CNP. The digestibility coefficients of DM and CP increased significantly, while EE decreased (*P* < 0.05) when birds were fed 0.4 g CNP/kg of feed. Other parameters, such as the digestibility of CF, Ca, and P were not affected (*P* > 0.05) by supplementing birds with different levels of dietary CNP.

**Table 4.** Effects of dietary CNP supplementation on digestibility of various nutrients in broilers at 35 to 38 d of age

Attributes (%)	CNP (g/kg diet)				SEM	*P*-value
	0	0.2	0.3	0.4		
DM	73.51^b^	73.69^b^	74.12^b^	75.43^a^	0.252	0.015
CP	65.11^b^	65.83^b^	66.09^b^	68.34^a^	0.390	0.009
EE	77.63^a^	77.34^a^	76.81^ab^	75.47^b^	0.296	0.033
CF	18.25	18.53	19.35	19.61	0.242	0.143
Ca	49.73	49.87	50.21	50.44	0.369	0.918
P	55.37	55.73	56.84	57.18	0.314	0.119

Ca: calcium; CF: crude fiber; CNP: chitosan nanoparticles; CP: crude protein; DM: dry matter; EE: ether extract; P: phosphorous.

The means in the same column bearing different superscripts are significantly different at *P* < 0.05.

### Serum Biochemicals

Results of supplementation of CNP at various levels to broiler birds on serum biochemistry are displayed in Table 5. The albumin and TP were significantly (*P* < 0.05) increased by including dietary CNP at 0.4 g/kg compared to the un-supplemented group. Adding 0.3 and 0.4 g CNP/kg diet resulted in a significant (*P* < 0.05) reduction in broilers’ cholesterol, ALT, and AST levels compared to the control.

**Table 5.** Effects of dietary CNP supplementation on serum biochemicals of broilers at 42 d of age

Attributes	CNP (g/kg diet)^*^				SEM	*P*-value
	0	0.2	0.3	0.4		
TP (g/dL)	3.67^b^	3.71^b^	3.75^ab^	3.91^a^	0.034	0.044
Albumin (g/dL)	2.19^b^	2.20^b^	2.24^ab^	2.34^a^	0.021	0.042
Globulin (g/dL)	1.48	1.50	1.52	1.57	0.015	0.135
A:G ratio	1.48	1.47	1.47	1.49	0.010	0.919
Cholesterol (mg/dL)	164.11^c^	157.20^bc^	145.17^ab^	136.73^a^	3.147	0.002
Triglyceride (mg/dL)	97.82	96.34	93.53	90.48	2.175	0.618
ALT (U/L)	112.87^a^	109.12^a^	101.80^ab^	94.02^b^	2.681	0.048
AST (U/L)	17.30^a^	15.78^ab^	14.05^ab^	12.57^b^	0.659	0.045

A:G ratio: albumin: globulin ratio; ALT: alanine transaminase; AST: aspartate aminotransferase; CNP: chitosan nanoparticles; TP: total protein.

^*^The average of 5 pens of 2 birds per pen.

The means in the same column bearing different superscripts are significantly different at *P* < 0.05.

### Immune Response

Table 6 lists the results of immune reaction in broilers fed various amounts of CNP. The serum immunoglobulin levels of IgG and IgM were significantly (*P* < 0.05) elevated in birds in the groups administered with 0.3 and 0.4 g CNP/kg in feed compared to the basal group.

**Table 6.** Effects of dietary CNP supplementation on immune response of broilers at 42 d of age

Attributes	CNP (g/kg diet)				SEM	*P*-value
	0	0.2	0.3	0.4		
IgG (µg/mL)	286.19^b^	295.56^b^	314.31^a^	326.47^a^	4.493	0.001
IgM (µg/mL)	534.17^c^	547.82^bc^	557.46^ab^	572.56^a^	4.568	0.012
Anti-SRBC titer (log_2_)	5.24^c^	5.32^bc^	5.41^b^	5.60^a^	0.039	0.001

CNP: chitosan nanoparticles; IgG: immunoglobulin G; IgM: immunoglobulin M, SRBC: sheep red blood cells.

The means in the same column bearing different superscripts are significantly different at *P* < 0.05.

### Microbial Load

Table 7 illustrates the influence of supplementing different levels of CNP on the caecal microbial load of broilers. The total bacteria and coliform counts were lower in birds-fed diets supplemented with 0.3 and 0.4 g/kg CNP compared to the control. Additionally, the *lactobacilli* number increased by incorporating 0.4 g/kg CNP in feed compared to other CNP groups and the control.

**Table 7.** Effects of dietary CNP supplementation on caecal microbial load of broilers

Attributes (CFU/g)	CNP (g/kg diet)				SEM	*P*-value
	0	0.2	0.3	0.4		
TBC	8.93^a^	8.89^ab^	8.81^bc^	8.76^c^	0.021	0.012
*Coliforms*	6.33^a^	6.28^ab^	6.23^b^	6.15^c^	0.019	<0.001
*Lactobacilli*	4.86	4.88	4.89	4.93	0.012	0.246

CNP: chitosan nanoparticles; TBC: total bacterial count.

The means in the same column bearing different superscripts are significantly different at *P* < 0.05.

### Carcass Traits

The impact of CNP supplementation on broiler carcass traits is outlined in Table 8. The dressing percentage and breast weight significantly improved (*P* < 0.05) by adding 0.4 g/kg of CNP to the diet. There was no significant difference between the treated and untreated groups in the heart, liver, and gizzard weight. Additionally, broilers fed with 0.3 and 0.4 g of CNP/kg in their basal diets had significantly lower abdominal fat than the control birds.

**Table 8.** Effects of dietary CNP supplementation on carcass attributes of broilers

Attributes	CNP (g/kg diet)				SEM	*P*-value
	0	0.2	0.3	0.4		
Live weight (g)	1,955.48^b^	1,969.36^b^	1,983.18^b^	2,019.67^a^	6.884	0.001
Dressing (%)^*^	71.21^b^	71.87^b^	72.24^ab^	73.65^a^	0.313	0.027
Breast weight (%)^*^	31.03^b^	31.24^b^	31.53^b^	32.98^a^	0.253	0.014
Thigh weight (%)^*^	27.63	27.85	28.09	28.53	0.188	0.197
Heart weight (%)^*^	0.44	0.45	0.44	0.46	0.009	0.916
Liver weight (%)^*^	2.46	2.45	2.48	2.47	0.014	0.880
Gizzard (%)^*^	1.87	1.88	1.88	1.90	0.011	0.885
Abdominal fat (%)^*^	1.18^a^	1.16^ab^	1.15^b^	1.13^b^	0.006	0.015

CNP: chitosan nanoparticles.

^*^Percent BW.

The means in the same column bearing different superscripts are significantly different at *P* < 0.05.

### Meat Quality

The impacts of CNP on the breast meat quality of broilers are presented in Table 9. A 0.4 g CNP/kg dose in the feed of broilers resulted in a significant (*P* < 0.05) increase in WHC and a reduction in DL) compared to the control group. TTBARS significantly declined (*P* < 0.05) by incorporating 0.4 g/kg CNP in broiler feed compared to other treated groups and the control. Regarding meat antioxidant parameters, birds’ ABTS and DPPH values increased by supplementing CNP at various levels compared to control birds.

**Table 9.** Effects of dietary CNP supplementation on meat quality of broilers

Attributes	CNP (g/kg diet)				SEM	*P*-value
	0	0.2	0.3	0.4		
pH	5.99^a^	5.94^a^	5.88^ab^	5.75^b^	0.030	0.015
WHC (%)	53.08^b^	53.29^b^	54.02^b^	56.13^a^	0.425	0.018
DL (%)	3.18^a^	3.15^a^	3.07^ab^	2.97^b^	0.026	0.008
Color coordinates of meat						
L*	58.33	57.54	58.27	59.45	0.906	0.919
a*	5.76	5.85	5.87	5.79	0.118	0.989
b*	11.37	11.29	11.33	11.44	0.050	0.790
TBARS (mg MDA/kg)	0.46^a^	0.44^a^	0.41^ab^	0.35^b^	0.015	0.032
ABTS (%)	86.14^c^	85.21^c^	89.29^b^	92.88^a^	0.797	<0.001
DPPH (%)	20.84^b^	21.10^b^	21.84^b^	23.54^a^	0.354	0.016

CNP: chitosan nanoparticles; WHC: water holding capacity; DL: drip loss; L*: lightness; a*: redness; b*: yellowness; TBARS: thiobarbituric acid reactive substance; ABTS: 2,2-azinobis (3-ethylbenzothiazoline- 6-sulfonic acid); DPPH: 2,2-diphenyl-1-picrylhydrazyl.

The means in the same column bearing different superscripts are significantly different at *P* < 0.05.

## Discussion

Various feed additives are implied to boost growth performance, feed efficiency, and sustain birds’ health. Nanotechnology can improve the efficacy and availability of raw substances. In the present study, CNP supplementation improved the BW, BWG, and feed efficiency at 0.4 g/kg in the diet of broiler birds. Shi et al. (2005) also documented an improvement in growth and feed efficiency following supplementation of chitosan at 0.5 or 1.0 g/kg of diet. Similar findings were revealed by Nuengjamnong and Angkanapron (2017) by including chitosan in a 2 g/kg diet of chicken. An increase in BW, BWG with better FCR in broiler birds fed chitosan oligosaccharides in their basal diet has earlier been reported (Li et al., 2019; Ahmed et al., 2021). In contrast, Khambuali et al. (2009) detected no difference in broilers fed 0.6 g/kg chitosan feed efficiency up to 7 wk of age. The growth-promoting impact of chitosan has been attributed to better feed digestibility and utilization, stimulation of the secretions of growth factors such as growth hormones and insulin-like growth factor I (Chen et al., 2009), improvement in gut morphology and microbial community (Hu et al., 2020; Yang et al., 2022).

The digestibility trials help us to know about the digestion, absorption, and utilization of nutrients in the digestive tract. The digestibility coefficients in our study revealed a significant improvement in the digestibility of CP and DM in birds received 0.4 g CNP/kg of feed compared to control and other dietary treatments. Huang et al. (2005) documented an improvement in the ileal digestibility of broiler birds fed different levels of chitosan oligosaccharides up to 150 mg/kg diet. However, EE’s digestibility decreased significantly when broilers were supplemented with dietary CNP at 0.4 g/kg. Chitosan interacts with the hydrochloric acid of the stomach. It forms a cationic gel (Ahmadi et al., 2015) which has a strong affinity for anionic molecules like fats, fatty acids, and other fats, thereby decreasing the fat absorption in the intestine by limiting micelle formation during lipid digestion (De Aguiar Vallim et al., 2013; Liu et al., 2021). These statements support our findings, wherein the digestibility of EE was decreased by augmenting the concentration of CNP in broilers’ diets.

The present study revealed a significant improvement in dressed and breast meat yield of birds-fed diets added with CNPs at 0.4 g/kg diet related to control and other CNP-supplemented groups. Moreover, the weight of the thigh, heart, gizzard, and liver were not affected by the incorporation of CNP at various levels compared to the control. The abdominal fat yield was significantly decreased dose-dependent on increasing the concentration of CNP in the diet. Similar to our data, Lan et al. (2023) monitored an increase in the eviscerated and breast meat yield while a linear decline in abdominal fat yield on adding chitosan oligosaccharides up to 0.9 g/kg of diet. Some studies indicated no effects of chitosan on carcass yield and cut-up parts but decreased abdominal fat content (Arslan and Tufan, 2018; Wang et al., 2022). This reduction in the abdominal fat content could be attributed to the fact that chitosan inhibits fat deposition by decreasing the absorption of lipids in GIT or by reducing liver lipogenesis (Zhou et al., 2009; Li et al., 2016; Chiu et al., 2017). The decreased abdominal fat deposition in CNP groups not only increases the market value of broilers but also prevents the economic losses incurred during the processing of effluent (Jayathilakan et al., 2012; Liu et al., 2019).

In the present study, broilers fed diets supplemented with CNP showed an enhancement in the immune response compared to control birds. The immune-stimulatory influence was examined by increased serum concentration of immunoglobulins (IgG and IgM) and antibody titer against the Newcastle disease (**ND**) virus. Our outcomes are consistent with Huang et al. (2005) and Deng et al. (2008), who informed an improved IgG and IgM concentration in birds fed chitosan oligosaccharides in the diet compared to the control birds. Jan et al. (2012) documented that chitosan, and its derivatives could enhance immunity in broilers by increasing antibody titer against ND virus and CD4^+^T-cell levels. Moreover, Youssef et al. (2023) observed that supplementing chitosan oligosaccharides up to 0.5 g/kg diet in laying hens improved serum IgG and IgM levels and antibodies against avian influenza and ND virus. The immunostimulatory effect of chitosan may be due to reactive amino and hydroxyl functional groups that trigger the immune system for the production of antibodies, initiating the release of chemokines, cytokines, activating macrophages, etc., thus promoting immunity (Fong and Hoemann, 2018).

The serum biochemical profile indicates a bird’s nutritional, physiological, and pathological levels. The serum biochemical profile of a bird changes with age and during certain situations like nutrition, diseases, etc. In our study, the levels of TP and its fractions (albumin and globulin) augmented in a dose-dependent manner and were significantly higher in the 0.4 g CNP/kg supplemented group with values falling within the normal (2.5 to 4.5 g/dL) range (Campbell, 2004). The results agree with those of Li et al. (2007) and Ayman et al. (2022), who stated that higher values of these parameters include chitosan oligosaccharides in broilers. Moreover, Ali et al. (2023) observed increased parameters in heat-stressed birds fed CNP at a lower dose of 50 mg/L in drinking water than the control. These variations might be due to species or breed under study and the medium through which CNP was provided to the birds (feed or water). The serum TP levels depict the protein reserve in the body (Azis et al., 2012). Proteins are required for growth and other functions like immunity and disease prevention; low levels indicate liver damage or other diseases. The increased levels of TP, albumin, and globulin in our study might be due to better absorption and digestion of nutrients and subsequently increased immune response in birds by supplementing CNP in their diet. However, some studies revealed no change in the albumin and TP values in birds-fed diets enriched with chitosan (Zhou et al., 2009; Lebiebicier and Aydogan, 2018). Compared to the control, total cholesterol levels significantly declined, with no change in the value of triglycerides.

Compared to the control, the total cholesterol was reduced by all levels of CNP supplementation, with the lowest reduction in the 0.4 g/kg CNP-supplemented group. Our results coincide with others (Osman et al., 2010; Tufan and Arsalan, 2020; Ayman et al., 2022), who reported the hypocholesterolemic impact of chitosan when used as a feed additive in the diet of broilers. Further, the level of triglycerides was not significantly affected by CNP dietary inclusion in the existing experiment. Disagreeing with our outcomes, Ayman et al. (2022) observed lower levels of triglycerides on supplementation of chitosan oligosaccharides in broiler diet. Moreover, some findings revealed no change in the lipid profile following supplementation of chitosan in the diet of broilers (Leblebicier and Aydogan, 2018; Jasim and Al-Naif, 2021). The hypocholesterolemic effect observed in our study could be due to increased viscosity of digestive content of the gastrointestinal tract that interferes with lipid absorption and/or greater affinity of chitosan for anionic molecules like lipids, bile salts, limiting fat digestion and absorption in intestines (De Aguiar Vallim et al., 2013; Liu et al., 2021).

The activity of liver enzymes like ALT and AST is an important indicator of a healthy liver (Ambrosy et al., 2015). Our paper revealed a significant decline in ALT and AST levels by adding CNP beyond 0.2 g/kg diet. Our results agree with Chang et al. (2020), who documented a decline in serum ALT and AST levels in broilers under elevated temperatures when chitosan oligosaccharides were supplemented in the diet. The chitosan oligosaccharides lowered the serum AST and ALT levels in rats by masking the harmful effect of lipopolysaccharides on the liver (Qiao et al., 2011). Contrary to our results, Jasim and Al-Naif (2021) observed no change in ALT and AST serum levels by chitosan supplementation to the basal diet. Similarly, Ayman et al. (2022) reported no change in serum ALT but a strengthening in the levels of AST in birds fed chitosan oligosaccharides in their diet.

Poultry health is closely associated with gut microbiome composition and diversity. Microbiota of GIT includes both beneficial bacteria (like *lactobacilli*) and pathogenic bacteria (like *coliforms*) (Li et al., 2009). The microbiome of GIT is involved in protection against pathogens, nutrient production and absorption, immune system maturation, and birds’ overall performance (Stanely et al., 2014). Poor intestinal health results in the malabsorption of nutrients and reduced growth in birds (Bailey, 2010). Our study revealed a decrease in the TBC and coliform count in dietary CNP-supplemented groups compared to the control, with a maximum reduction in the 0.4 g CNP/kg diet group compared to the control.

In contrast, LAB count did not reveal any significance between the control and all other dietary treatments. Inconsistent with our results, Nuengjamnong and Angkanapron (2017) reported a significant reduction in the *coliform* count in birds-fed diets with supplemental chitosan at 1 to 2 g/kg. Furthermore, Ali et al. (2023) reported a decreased count of *coliforms* with an increase in the *lactobacilli* population in the cecum of broilers fed dietary CNP at 50 mg/kg diet. The decreased coliform count could be due to forming a biofilm-like structure in *coliforms* and the promoting colonization of GIT by *lactobacilli* (Xu et al., 2020).

A substantial rise in pH value of breast meat was monitored in 0.4 g CNP/kg group when compared with the basal diet and other CNP groups, but the values were well within the range for normal quality meat. The pH value of valuable quality broiler meat has been stated to range from 5.9 to 6.2 (Ristc and Damme, 2010). One of the critical attributes employed by the meat trade in the judgment of meat quality is its pH value (Bianchi et al., 2005). Increased pH directly depends on meat quality features like improved WHC and decreased DL, etc. Higher WHC increases tenderness, juiciness, and appearance and improves meat quality (Mir et al., 2017). Our study reported an increase in the value of WHC with a corresponding decrease in the DL values upon increasing the supplementation of CNP in the broiler birds compared to the control. Similar to our outcomes, Lan et al. (2023) documented a significant increase in breast meat’s pH value and a diminished DL value on increasing the supplementation of chitosan oligosaccharides up to 0.9 g/kg of diet.

The coloration of fresh poultry meat is a significant issue in consumer preference for meat (Kuttappan et al., 2012). Meat color is associated with its freshness, acceptability, and attractiveness by consumers (Mir et al., 2017). Broilers supplemented with varying concentrations of CNP in the diet were observed to maintain color coordinates of breast meat within the normal range of acceptability by the consumers. Contrary to our results, Lan et al. (2023) examined a significant decrease in the lightness (*L*) of meat with no effect on the redness (*a*), and yellowness (*b*) of meat following chitosan supplementation in broilers. The MDA content (by TBARS) of meat indicates the degree of lipid peroxidation. Poultry meat contains many highly unsaturated fatty acids, making it more susceptible to oxidative deterioration than other meat categories. The TBARS method measures the extent of rancidity or souring that arises from fat and fatty parts of meat undergoing oxidation. It determines the level of oxidative damage by assessing the formation of substances such as MDA (a lipid peroxidation marker) (Tekce et al., 2020).

Tests were also performed to determine the antioxidant potential of broiler meat by estimating DPPH and ABTS values. In the current study, a reduction in the level of TBARS, while an increase in the activity of ABTS and DPPH was observed in the chicken in a dose-dependent manner following supplementation with CNP up to 0.4 g/kg of diet. Inconsistent with our results, Lan et al. (2023) documented a decline in the MDA content and augmented the activity of DPPH and ABTS values by dietary chitosan oligosaccharide supplementation in frizzle chicken. Moreover, Chang et al. (2020) observed a reduced lipid peroxidation (lower MDA content) and heightened muscle antioxidant status in heat-stressed broilers fed chitosan oligosaccharide in the diet.

## Conclusions

The supplementation of chitosan nanoparticles (CNP) at 0.4 g/kg diet enhanced the broilers’ performance and nutrient digestibility. Further, adding dietary CNP at 0.4 g/kg positively impacts serum cholesterol, liver function, immune response, and microbial load of birds. In addition, the carcass and meat quality attributes were improved by CNP inclusion in the diet at 0.4 g/kg. Hence, CNP supplementation at a 4 g/kg dose rate is recommended as a feed additive in broiler chickens.
